# European Stroke Organisation guidelines on stroke in women: Management of menopause, pregnancy and postpartum

**DOI:** 10.1177/23969873221078696

**Published:** 2022-03-29

**Authors:** Christine Kremer, Zuzana Gdovinova, Yannick Bejot, Mirjam R Heldner, Susanna Zuurbier, Silke Walter, Avtar Lal, Corina Epple, Svetlana Lorenzano, Marie-Luise Mono, Theodore Karapanayiotides, Kailash Krishnan, Dejana Jovanovic, Jesse Dawson, Valeria Caso

**Affiliations:** 1 Neurology Department, Clinical Sciences Lund University, Skåne University Hospital, Malmö, Sweden; 2 Neurology Department, Faculty of Medicine, Pavol Jozef Safarik University Košice, Košice, Slovakia; 3 Dijon Stroke Registry, Pathophysiology and Epidemiology of Cerebro-Cardiovascular diseases (PEC2), University of Burgundy, University Hospital of Dijon, Dijon, France; 4 Department of Neurology, Inselspital, 27252 University Hospital and University of Bern, Bern, Switzerland; 5 Department of Neurology, 563540 Amsterdam University Medical Centers, Amsterdam, Netherlands; 6 Department of Neurology, 39072 Saarland University, Homburg, Germany; 7 European Stroke Organisation (ESO), Basel, Switzerland; 8 Department of Neurology, 39790 Klinikum Hanau, Hanau, Germany; 9 Department of Human Neurosciences, 9311 Sapienza University of Rome, Rome, Italy; 10 Department of Neurology, Municipal Hospital Waid und Triemli, Zürich, University Hospital and University of Bern, Bern Switzerland; 11 2nd Department of Neurology, School of Medicine, Faculty of Health Sciences, 37788 Aristotle University of Thessaloniki, Thessaloniki, Greece; 12 Stroke, Department of Acute Medicine, Queens Medical Centre, Nottingham University Hospitals NHS Trust, Nottingham, UK; 13 Department of Emergency Neurology, Neurology Clinic, Medical Faculty, University Clinical Center of Serbia, University of Belgrade, Belgrade, Serbia; 14 Institute of Cardiovascular and Medical Sciences, College of Medical, Veterinary & Life Sciences, 236381 University of Glasgow, Glasgow, United Kingdom; 15 Stroke Unit, Santa Maria della Misericordia Hospital, University of Perugia Perugia, Italy

**Keywords:** Stroke, guidelines, women, menopause, pregnancy, postpartum

## Abstract

Pregnancy, postpartum and menopause are regarded as periods women are more vulnerable to ischaemic events. There are conflicting results regarding stroke risk and hormone replacement therapy (HRT) during menopause. Stroke in pregnancy is generally increasing with serious consequences for mother and child; therefore, recommendations for acute treatment with intravenous thrombolysis (IVT) and/or mechanical thrombectomy (MT) are needed. The aim of this guideline is to support and guide clinicians in treatment decisions in stroke in women. Following the “Grading of Recommendations and Assessment, Development and Evaluation (GRADE)” approach, the guidelines were developed according to the European Stroke Organisation (ESO) Standard Operating Procedure. Systematic reviews and metanalyses were performed. Based on available evidence, recommendations were provided. Where there was a lack of evidence, an expert consensus statement was given. Low quality of evidence was found to suggest against the use of HRT to reduce the risk of stroke (ischaemic and haemorrhagic) in postmenopausal women. No data was available on the outcome of women with stroke when treated with HRT. No sufficient evidence was found to provide recommendations for treatment with IVT or MT during pregnancy, postpartum and menstruation. The majority of members suggested that pregnant women can be treated with IVT after assessing the benefit/risk profile on an individual basis, all members suggested treatment with IVT during postpartum and menstruation. All members suggested treatment with MT during pregnancy. The guidelines highlight the need to identify evidence for stroke prevention and acute treatment in women in more vulnerable periods of their lifetime to generate reliable data for future guidelines.

## Introduction

Pregnancy, postpartum and menopause are periods of life with an increased vulnerability for stroke in women. Treatment with hormone replacement therapy (HRT) in postmenopausal women and associated stroke risk has led to conflicting results.^1,2^ In contrast, some studies reported a higher stroke risk under and after HRT while other did not. There is scarce information about the risk of intracerebral haemorrhage in women taking HRT and it is unclear whether HRT should be recommended in postmenopausal women to reduce the risk for stroke. Several large randomized clinical trials reported the outcome of postmenopausal women treated with different types of HRT, for example, oestrogen, medroxyprogesterone and selective oestrogen receptor modulators. Here, we summarize the available evidence on the risk for developing an ischaemic or haemorrhagic stroke when being treated with HRT. Special consideration is given to the subgroup of patients with severe and fatal stroke.

By presenting the available data, this guideline shall support the evidence-based treatment of postmenopausal women.

Acute ischaemic stroke during pregnancy is a rare but serious complication. Stroke occurs in 34 of every 100,000 deliveries, at least one-half of pregnancy-related strokes are likely to be of the ischaemic stroke subtype.^3^ Moreover, stroke in pregnancy is increasing^4^, likely due to older maternal age at birth.^5^

Only recently, pregnancy was removed from the list of contraindications for intravenous thrombolysis (IVT) in the 2018 American Heart Association/American Stroke Association Stroke Guidelines for the early management of patients with acute ischaemic stroke^6^, and the 2018 Canadian Stroke Best Practice Consensus Statement.^7^

During pregnancy and in postpartum period, haemodynamic changes, the hypercoagulable state, hypertensive disorders of pregnancy and their complications contribute to the increased risk of stroke.^7,8^ On the other hand, acute treatment of stroke is associated with the risk of bleeding, and current recommendations for acute stroke treatment do not apply to women in puerperium; therefore, recommendations for acute treatment with IVT and/or mechanical thrombectomy (MT) are needed. Intravenous thrombolysis with alteplase is the only approved systemic reperfusion treatment for patients with acute ischaemic stroke^9^, and MT is recommended for patients with large vessel occlusion.^10^ Pregnant and postpartum women were excluded from all randomized controlled trials (RCTs) on acute stroke reperfusion/recanalization treatments, which led to a lack of evidence on potentially beneficial therapies in this patient population. For this reason, most pregnant or postpartum women with ischaemic stroke, otherwise potentially eligible, do not receive reperfusion therapy.

Women receiving IVT during menstruation might show an increased uterine bleeding risk. During menstruation haemostasis is not only regulated by platelet activation and aggregation and deposition of fibrinogen but also by prostaglandins, hormones and myometrial contraction.^11^ There is continued uncertainty regarding safety of IVT or/and MT of women during menstruation.^12^

The ESO-guideline group on stroke in women prepared this guideline module based on GRADE methodology and ESO Standard Operating Procedure^13,14^ to guide clinicians in their everyday clinical practice.

## Methods

These guidelines were initiated by the European stroke organisation (ESO) and the ESO Guidelines Committee. A Module Working Group (MWG) was established, consisting of Christine Kremer (CK), Zuzana Gdovinova (ZG), Svetlana Lorenzano (SL), Yannick Bejot (YB), Mirjam Heldner (MH), Susanna Zuurbier (SZ), Silke Walter (SW), Corina Epple (CE), Marie-Luise Mono (MLM), Jesse Dawson (JD), Theodore Karapanaytoides (TK), Dejana Jovanovic (DJ), Valeria Caso (VC). The composition of this group was approved by the ESO Guidelines Board and the ESO Executive Committee, based on a review of the intellectual and financial disclosures of the proposed members.

**Figure 1.**
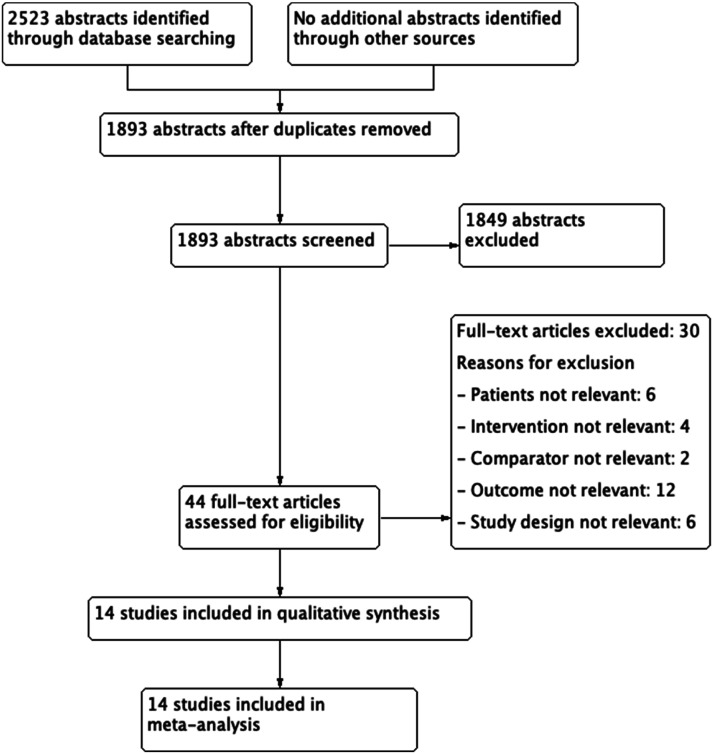
Prisma flow diagram on Hormone replacement therapy (HRT) and Stroke Risk.

**Figure 2.**
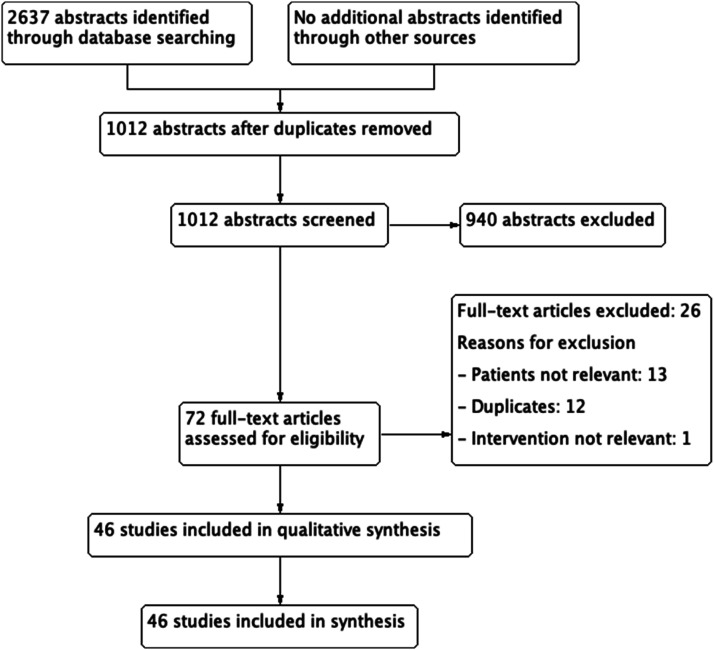
PRISMA flow diagram on intravenous thrombolysis (IVT) and ET during pregnancy, postpartum and menstruation.

As described previously, the guidelines were developed using GRADE methodology^15^ and the ESO Standard Operating Procedure.^13,14^ In brief, the MWG developed a list of topics and corresponding outcomes of clinical interest. The ESO guideline working group identified and discussed the two issues according to clinical importance, lack of data and current existing guidelines. The outcomes were rated as critical, important or of limited importance according to GRADE criteria by the MWG.^13,15^ (Supplementary Table 1) A series of PICO (Population, Intervention, Comparator, Outcome) questions were developed and approved by the ESO Guidelines board and the ESO Executive Committee. For each question, systematic searches of the MEDLINE, EMBASE, CINAHL and SCOPUS databases, covering the period from the inception of each database to 2020, were conducted by the ESO Guidelines methodologist, Avtar Lal (AL). AL, CK and VC agreed on the search terms for each PICO question. Potentially eligible RCTs, meta-analyses and observational studies were identified, and citations were loaded on COVIDENCE software. Titles and abstracts of publications were identified from the searches, and potentially relevant studies were assessed for each chapter in duplicate and independently by members of each subgroup according to pre-defined inclusion/exclusion criteria (first level screening). Full texts were downloaded onto the software and assessed following the same inclusion and exclusion criteria (second level screening) (Figure 1 and Figure 2).

A group of MWG members (a ‘PICO group’) was formed to evaluate the available evidence for each question. PICO1: MH, SW, SZ, CE, MLM and CK; PICO 2: ZG, SL, YB, JD, TK, DJ and VC. The risk of selection, performance, detection, attrition and reporting biases in each randomized trial was assessed using the Cochrane Collaboration’s tool^16^, and heterogeneity across studies was evaluated using Cochran’s Q (reported as a p-value) and I^2^ statistics.^17^ Meta-analyses were performed by using the RevMan software, using a Random-effects model. Odds ratio (OR) were calculated for dichotomous variables and mean differences (MD) for continuous variables, along with their 95% confidence interval (CI). A value of *P* < .05 was considered for statistical significance. Any heterogeneity across studies was assessed using the *I* ^*2*^ statistic, and heterogeneity was classified as moderate (*I* ^*2*^  ≥  30%), substantial (*I* ^*2*^  ≥  50%) or considerable (*I* ^*2*^  ≥  75%). The heterogeneity was checked by a high value of *I* ^*2*^ and *P* < .05. For each PICO question and outcome, the quality of evidence was rated using the GRADEpro Guideline Development Tool (McMaster University, 2015; developed by Evidence Prime, Inc.) as high, moderate, low or very low^13^ The relevant PICO group was responsible for analysing the available data and formulating an evidence-based recommendation according to the GRADE evidence profiles and the ESO standard operating procedure. Expert Opinion statements, based on voting by all MWG members, was presented where the PICO group considered that not enough evidence was available to provide evidence-based recommendations for situations where practical guidance was needed for everyday clinical practice. Importantly, these Expert Opinions should not be regarded as evidence-based recommendations since they only reflect the opinion of the MWG.

The Guideline document was reviewed several times by all MWG members and modified using a Delphi approach until consensus was reached. Two external reviewers, and ESO Guideline Board, and the Executive Committee members, reviewed and approved the paper.

## PART 1: Hormone replacement therapy (HRT) and stroke risk

### PICO 1.1: In postmenopausal women, does HRT compared to non-prior HRT reduce the risk of ischaemic stroke in primary prevention?

#### Analysis of current evidence

The analysis included six randomized controlled clinical trials (three trials on HRT and three on receptor modulators). Data from 29,233 patients with HRT and 15,463 control patients were analysed.^18–23^

The studies showed that HRT did not reduce the risk of ischaemic stroke [odds ratio (OR) 0.97, 95% CI 0.66–1.41, *P* = 0.86, *I* ^2^ = 65%].

The overall quality of evidence was rated as very low, with a serious risk of inconsistency indicated by *I* ^2^ ≥ 65%. Due to the overall low number of studies available and an overall strongly suspected publication bias, a serious risk of indirectness and imprecision indicated by wide confidence intervals was present (Table 1).

**Table 1.** Grade evidence profile table for Population, Intervention, Comparator, Outcome (PICO) 1.1.

Certainty assessment							No of patients		Effect		Certainty	Importance
№ of studies	Study design	Risk of bias	Inconsistency	Indirectness	Imprecision	Other considerations	Hormone replacement therapy	Control	Relative (95% CI)	Absolute (95% CI)		
Ischaemic stroke												
6	Randomized trials	Not serious	Serious^a^	Serious	Serious^b^	Publication bias strongly suspected^c^	286/29233 (1.0%)	194/15463 (1.3%)	OR (0.66 to 1.41)	0 fewer per 1,000 (from 4 fewer to 5 more)	⊕○○○ VERY LOW	CRITICAL
Ischaemic stroke – HRT												
3	Randomized trials	Not serious	Not serious	Not serious	Not serious	Publication bias strongly suspected^c^	200/17922 (1.1%)	140/10726 (1.3%)	OR 1.36 (1.09 to 1.69)	5 more per 1,000 (from 1 more to 9 more)	⊕⊕⊕○ MODERATE	CRITICAL
Ischaemic stroke – Receptor modulator												
3	Randomized trials	Not serious	Not serious	Not serious	Not serious	Publication bias strongly suspected^c^	86/11311 (0.8%)	54/4737 (1.1%)	OR 0.66 (0.47 to 0.93)	4 fewer per 1000 (from 6 fewer to 1 fewer)	⊕⊕⊕○ MODERATE	CRITICAL

CI: Confidence interval; OR: Odds ratio HRT: Hormone replacement therapy.

^a^I2 ≥ 65%.

^b^Wide confidence intervals

^c^6 or less studies reported this outcome.

#### Additional information

**Figure 3.**
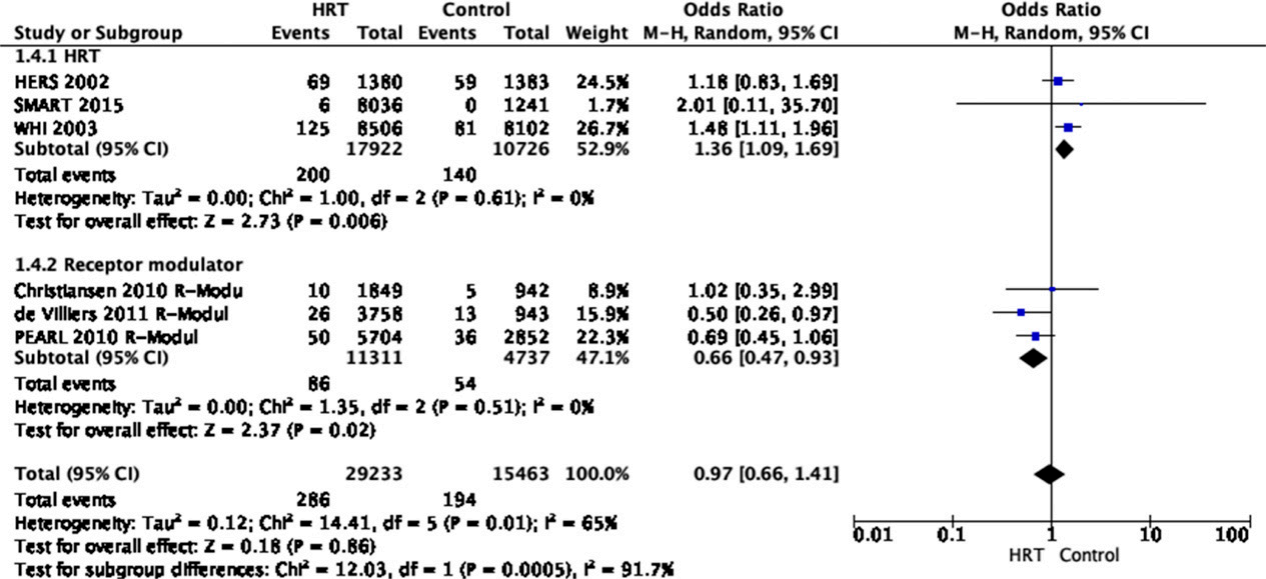
Pooled odds ratio for ischaemic stroke in menopausal women treated with HRT versus non-prior HRT.

Two RCTs analysed treatment with hormone replacement with conjugated equine oestrogen 0.625 mg/d and medroxyprogesterone acetate 2.5 mg/d^18,19^, and a pooled meta-analysis of 5 RCTs added information about treatment with different dosages of tissue-selective oestrogen complex pairs conjugated oestrogens plus the selective oestrogen receptor modulator bazedoxifene.^20^ These studies analysed 17,922 postmenopausal HRT patients versus 10,726 control patients. The summarised analysis of these studies showed an increased risk of HRT treated women for developing an ischaemic stroke [odds ratio (OR) 1.36, 95% CI 1.09–1.69, *P* = .006, *I* ^2^ = 0%]. The quality of evidence was rated as moderate, with no serious risk of bias, inconsistency, indirectness or imprecision but with a strongly suspected publication bias due to the overall low number of studies available. In addition, two trials were performed before 2000 and all trials recruited healthy (no previous stroke) women. (Figure 3)

The first trial investigating the risk of stroke with HRT analysed data collected from the Heart & Estrogen-progestin Replacement Study (HERS) study, designed as a secondary prevention trial of coronary heart disease. One thousand three hundred eighty postmenopausal women with coronary heart disease were randomly assigned to treatment with conjugated equine oestrogen (0.625 mg/d) and 2.5 mg/d medroxyprogesterone acetate, and 1383 women were assigned to placebo. Participants were recruited between 1993 and 1994 in 20 centres in the USA. The results showed no significant difference in the number of ischaemic strokes in both groups OR 1.18, 95% CI 0.83–1.67 after a mean follow-up of 2, 4 years.^18^

The second study is a pooled analysis of 5 phase III studies – Selective estrogens, Menopause, And Response to Therapy (SMART trials)^20^ performed to improve the understanding of vascular safety of a combined HRT and receptor modulator treatment. Four different pooled groups were analysed: 1585 women randomly assigned to 0.45 mg tissue-selective oestrogen complex pairs conjugated oestrogens (CE) plus 20 mg bazedoxifene, 1583 women allocated to 0.625 mg CE plus 20 mg bazedoxifene, 4868 women assigned to any dosage of CE and bazedoxifene and 1241 women treated with placebo. Study duration varied between the individual SMART trials, and safety data was collected for up to 2 years in the pooled analysis. Healthy participants were recruited between 2002 and 2011 in clinical centres in the USA, South America, Europe and Australasia. The results of the pooled analysis showed no significant difference in the number of ischaemic strokes in all groups both groups: 0.45 mg CE/20 mg bazedoxifene and 0.625 mg CE/20 mg bazedoxifene: relative risk (RR) 0.9, 95% CI 0.2–4.8; a group with any dosage CE and bazedoxifene: RR 0.5, 95% CI 0.1–2.6.^20^

The third study, the Women’s Health Initiative (WHI) trial, was a randomized, double-blind study designed as a large platform trial investigating different strategies for controlling common causes of morbidity and mortality in postmenopausal women. The HRT study arm involved 8506 women randomly assigned to 0.625 mg conjugated equine oestrogen and 2.5 mg/day of medroxyprogesterone acetate/day and 8102 women to placebo. Healthy participant recruitment was initiated in 1992 at 40 clinical centres in the USA. The results showed no significant difference in the number of ischaemic strokes in both groups [hazard ratio (HR) 1.44, 95% CI 1.09–1.9].^20^

Three RCTs analysed treatment with the receptor modulators bazedoxifen^21,22^, raloxifene^21^ and lasofoxifen.^23^ In these studies, 11,311 receptor modulator-treated patients versus 4737 control patients were analysed. The summarised analysis of these studies showed a reduced risk for an ischaemic stroke [OR 0.66, 95% CI 0.47–0.93, *P* = 0.02, *I* ^2^ = 0%]. The quality of evidence was rated as moderate, with no serious risk of bias, inconsistency, indirectness or imprecision but with a strongly suspected publication bias due to the overall low number of studies available.

The first trial by Christiansen et al. was a randomized trial designed to assess the safety of bazedoxifen for the prevention and treatment of osteoporosis. The study contained 3 receptor modulator arms, and participants were randomly allocated to a study arm. One thousand eight hundred eighty-six women were assigned to 20 mg bazedoxifene, 1872 women to 40 mg bazedoxifene, 1849 women to 60 mg raloxifene and 1885 to placebo. The study was conducted at 206 centres worldwide within 3 years. The results showed no significant difference in the number of ischaemic strokes in the three groups [20 mg bazedoxifene: HR 0.9, 95% CI 0.37–2.22; 40 mg bazedoxifene: HR 1.2, 95% CI 0.54–2.87; 60 mg raloxifene: HR 1.0, 95% CI 0.42–2.44].^21^

The second study analysed a 2-year extension phase of the before mentioned trial, including assessing the risk of ischaemic stroke 5 years after randomization. Study groups in the extension phase were changed, and all participants on 40 mg bazedoxifene were transitioned to 20 mg after 4 years, while the raloxifene group was stopped after the 3-year core study. In line with the core study results, no significant difference in the risk of ischaemic stroke could be identified [20 mg bazedoxifene: HR 0.9, 95% CI 0.42–2.02; 40/20 mg bazedoxifene HR 1.1, 95% CI 0.52–2.35].^22^

The third study – Postmenopausal Evaluation and Risk Reduction With Lasofoxifene (PEARL) trial – was a randomized, double-blind study designed to assess the risk of non-vertebral fracture and oestrogen receptor-positive breast cancer under treatment with the receptor modulator lasofoxifene at 5 years. The study contained 2 groups with different lasofoxifene dosages, 0.25 mg/d and 0.50 mg/d and a placebo group. Two thousand eight hundred fifty-two women were allocated to each group. The participants were recruited between 2001 and 2003 at 113 clinical centres in 32 countries. The results did not show any significant difference in the numbers of participants developing ischaemic strokes [0.25 mg lasofoxifene: HR 0.66, 95% CI 0.39–1.11, *P* = .11; 0.5 mg lasofoxifene HR 0.72, 95% CI 0.43–1.19, *P* = .19].^23^

**Evidence-based Recommendation**

In postmenopausal women, we suggest against the use of HRT to reduce the risk of ischaemic stroke.

Quality of evidence: **Very low ⊕**

Strength of recommendation: **Weak against intervention ↓**

### PICO 1.2: In postmenopausal women, does HRT compared to non-prior HRT reduce the risk of haemorrhagic stroke in primary prevention?

**Figure 4.**
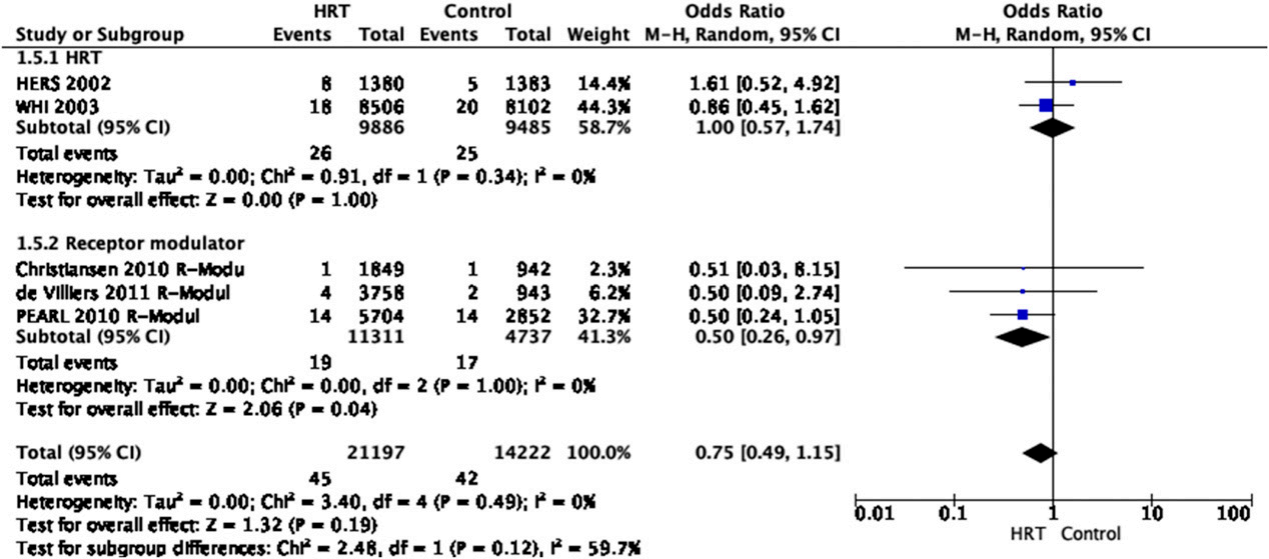
Pooled odds ratio for haemorrhagic stroke in menopausal women treated with HRT versus non-prior HRT.

Analysis of current evidence Five RCTs provide evidence for this question (two trials on HRT and three on receptor modulators).^18,19,21–23^ In total, data from 21,197 patients with HRT and 14,222 control patients from these five trials were analysed. The studies demonstrated that HRT non-significantly decreased the risk of haemorrhagic stroke (OR 0.75, 95% CI 0.49–1.15). The overall quality of evidence was rated as low, with a serious risk of publication bias and inconsistency, and imprecision. (Figure 4, Table 2)

**Table 2.** Grade evidence table for PICO 1.2.

Certainty assessment							№ of patients		Effect		Certainty	Importance
№ of studies	Study design	Risk of bias	Inconsistency	Indirectness	Imprecision	Other considerations	Hormone replacement therapy	Control	Relative (95% CI)	Absolute (95% CI)		
Haemorrhagic stroke												
5	Randomised trials	Not serious	Not serious	Not serious	Serious^a^	Publication bias strongly suspected^b^	45/21197 (0.2%)	42/14222 (0.3%)	OR (0.49 to 1.15)	1 fewer per (from 2 fewer to 0 fewer)	⊕⊕○○ LOW	CRITICAL
Haemorrhagic stroke – HRT												
2	Randomised trials	Not serious	Not serious	Not serious	Serious^a^	Publication bias strongly suspected^b^	26/9886 (0.3%)	25/9485 (0.3%)	OR 1.00 (0.57 to 1.74)	0 fewer per 1,000 (from 1 fewer to 2 more)	⊕⊕○○ LOW	CRITICAL
Haemorrhagic stroke – Receptor modulator												
3	Randomised trials	Not serious	Not serious	Not serious	Not serious	Publication bias strongly suspected^b^	19/11311 (0.2%)	17/4737 (0.4%)	OR 0.50 (0.26 to 0.97)	2 fewer per 1,000 (from 3 fewer to 0 fewer)	⊕⊕⊕○ MODERATE	CRITICAL

CI: Confidence interval; OR: Odds ratio; HRT. Hormone Replacement therapy.

^a^Wide confidence intervals.

^b^Five or less studies reported this outcome.

#### Additional information

The first trial on HRT that investigated the relationship between oestrogen plus progestin therapy and risk of stroke among postmenopausal women analysed data collected from the HERS, a secondary prevention trial in patients with coronary heart disease recruited patients between 1993 and 1994.^18^ Patients were randomized to HRT with conjugated equine oestrogen (0.625 mg/d) and 2.5 mg/d medroxyprogesterone acetate or placebo. This study reported that in postmenopausal women, HRT compared to non-prior HRT, non-significantly increased the risk of haemorrhagic stroke, OR 1.61, 95% CI: 0.52–4.92. In the WHI trial, participants were included between 1993 and 1998 and received 0.625 mg/d of conjugated equine oestrogen plus 2.5 mg/d of medroxyprogesterone acetate or placebo.^19^ They showed that in postmenopausal women, HRT compared to non-prior HRT, non-significantly decreased the risk of haemorrhagic stroke, OR 0.86, 95% CI: 0.45–1.62.

Heart & Estrogen-progestin Replacement Study included participants with established coronary heart disease with a high risk of stroke, while the WHI trial involved predominantly healthy women.^19^ These different study populations led to answers to the question of primary and secondary association of HRT and on stroke risk. Important to emphasise is that the WHI cohort (*n* = 16,608) was much larger than the HERS trial and that patients in the HERS and WHI trials were all recruited before 2000.

In three RCTs in postmenopausal women, the effect of receptor modulators to reduce the risk of haemorrhagic stroke in primary prevention has been investigated compared to non-receptor modulator therapy. In the first trial, Christiansen et al. investigated the effect of bazedoxifene, a novel selective oestrogen receptor modulator under development, to prevent and treat postmenopausal osteoporosis. Healthy postmenopausal osteoporotic women were randomized to daily doses of bazedoxifene 20 or 40 mg, raloxifene 60 mg or placebo for 3 years. The study demonstrated that in healthy postmenopausal osteoporotic women, a selective oestrogen receptor modulator non-significantly decreased the risk of haemorrhagic stroke (OR 0.51, 95% CI: 0.03–8.15)^21^. The second trial by De Villiers et al. showed that the results at 5 years were consistent with those seen at 3 years. During the 2-year study extension the raloxifene 60-mg treatment arm was discontinued after the 3-year database was finalized. Subjects receiving bazedoxifene 40 mg were transitioned in a blinded manner to bazedoxifene 20 mg after 4 years. The authors reported that in healthy postmenopausal osteoporotic women, a selective oestrogen receptor modulator non-significantly decreased the risk of haemorrhagic stroke (OR 0.50, 95% CI: 0.09–2.75) over 5 years of therapy, consistent with the findings at 3 years.^19,22^ In the third trial, PEARL, women with osteoporosis received lasofoxifene 0.25 mg/d, lasofoxifene 0.5 mg/d, or placebo for 5 years. This study showed that in postmenopausal women with osteoporosis, a selective oestrogen receptor modulator non-significantly decreased the risk of haemorrhagic stroke (OR 0.50, 95% CI: 0.24–1.05).^20,23^ In both trials, the risk of haemorrhagic stroke was decreased with a selective oestrogen receptor modulator. However, all trials included predominantly healthy postmenopausal women with osteoporosis. The overall quality of evidence was rated as low, with a serious risk of publication bias and inconsistency, and imprecision.

**Evidence-based Recommendation**

In postmenopausal women, we suggest against the use of HRT to reduce the risk of haemorrhagic stroke.

Quality of evidence: **Low ⊕⊕**

Strength of recommendation: **Weak against intervention** ↓

### PICO 1.3. In postmenopausal women with acute ischaemic stroke, does prior HRT compared with non-prior HRT impact functional outcome and mortality?

### And

### PICO 1.4. In postmenopausal women with acute haemorrhagic stroke, does prior HRT compared with non-prior HRT impact functional outcome and mortality?

#### Analysis of current evidence

No data is available on the functional outcome (using modified Rankin Scale (mRS) at 3 months) of postmenopausal women with acute stroke and with or without HRT.

Three RCTs provide evidence to stroke mortality of postmenopausal women with HRT without differentiation between haemorrhagic and ischaemic stroke^21,23,24^. In total, data from 11,824 patients with HRT and 5241 control patients were analysed. The studies demonstrated that HRT did not significantly impact stroke mortality [OR 1.24, 95% CI 0.7–2.19, *P* = 0.45, *I* ^2^ = 0%]. However, there was a tendency towards favouring the groups without HRT.

**Figure 5.**
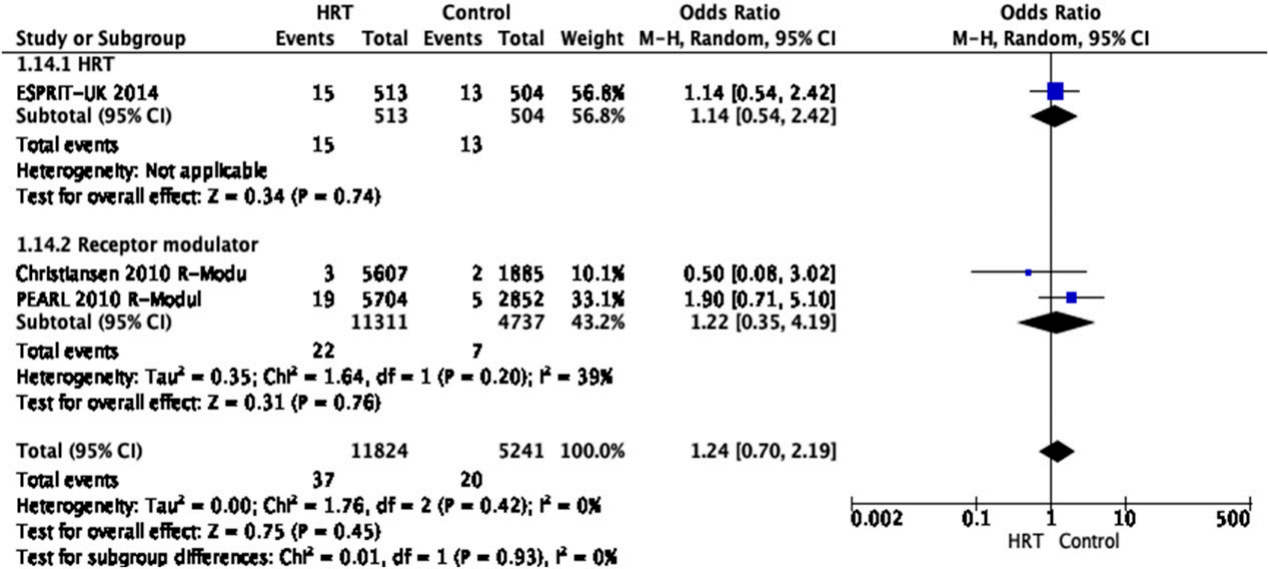
Pooled odds ratio for fatal stroke in menopausal women treated with HRT versus non-prior HRT.

The overall quality of evidence was rated as very low, with a serious risk of imprecision indicated by the very wide confidence intervals and an overall strongly suspected publication bias due to the overall low number of studies available. (Figure 5)

**Evidence-based Recommendation**

In postmenopausal women with acute stroke, we suggest against the use of HRT to reduce mortality.

Quality of evidence: **Very low ⊕**

Strength of recommendation: **Weak against intervention** ↓

## PART 2. Treatment of acute ischaemic stroke in pre-menopausal women (pregnancy, postpartum, and menstruation)

### PICO 2.1. In pregnant women with acute ischaemic stroke, does intravenous thrombolysis (IVT) improve outcome as compared to no IVT?

#### Analysis of current evidence

Currently, there are no RCTs on the use of acute stroke treatments with IVT in pregnant women.

#### Additional information

Only in the US Get With The Guidelines (GWTG) Stroke Registry, outcomes following IVT, catheter-based thrombolysis or MT or any combination of these treatments in pregnant/postpartum and non-pregnant/non-postpartum women were compared.^3^ There were similar rates of acute stroke reperfusion therapy in the pregnant or postpartum versus non-pregnant women (11.8% vs. 10.5%; *P* = 0.42). Pregnant or postpartum women were less likely to receive IVT monotherapy (4.4% vs. 7.9%; *P* = 0.03), primarily because of pregnancy and recent surgery. There was no difference in reperfusion rates with IVT/MT.

There were also substantial differences in demographics and baseline characteristics because pregnant/postpartum women were younger (median age, Interquartile range [IQR]: 31 [26–35] vs. 39 [33–42] years, *P* < 0.0001) and had a higher National Institute of Health Stroke Scale (NIHSS) score at baseline (median, [IQR]: 13 [8–16] vs. 9 [5–15], *P* = 0.01) than non-pregnant/non-postpartum patients. Outcomes in pregnant/postpartum women receiving reperfusion therapy were overall comparable to those observed in non-pregnant/non-postpartum women. A trend towards increased symptomatic intracranial haemorrhage in the pregnant/postpartum women was observed (7.5% [95% CI: 1.6–20.4%] vs. 2.6% [95% CI: 2.0–3.3%]; *P* = 0.06). However, there were no cases of major systemic bleeding or in-hospital deaths, and moderate rates of discharge to home (57.5% vs. 63.6%, *P* = 0.43) and of independent ambulation at discharge (55.9% vs. 64.1%, *P* = 0.33) were observed, similarly to their non-pregnant/non-postpartum counterparts. Of note, a prolonged length of hospital stay of > 4 days was more common in the pregnant or postpartum group (72% vs. 41.7%), with most of these patients being discharged home.^3^

Besides these data from the GWGT Stroke Registry, we found only single case reports in our search. Therefore, it should be considered that the risk of bias is high. Cases reported in abstract formats were not included. From 33 individual cases, 25 patients were treated with IVT alone, the remaining eight received combined treatment (IVT + MT)^25–53^ All patients had a neurological improvement on NIHSS score compared to baseline, with almost all of them achieving functional independence, except for only one patient – treated with IVT alone – who had a final NIHSS score of 14, however, improved compared to the admission score of 23 (Table 3). Alteplase was administered during all three trimesters. However, most patients had their stroke and acute treatment in the first trimester (13 patients; 10 patients in the second and third trimesters).

**Table 5.** Synoptic table of all recommendations.

Topic/PICO question	Recommendation	Expert consensus statement
1. Hormone replacement therapy (HRT) and stroke risk	In menopausal women we suggest against the use of HRT to reduce the risk of ischaemic stroke.	
1.1. In menopausal women, does HRT compared to non-prior HRT reduce the risk of ischaemic stroke?	Quality of evidence: Very low ⊕	
	Strength of recommendation: Weak against intervention ↓	
1.2 In menopausal women, does HRT compared to non-prior HRT reduce the risk of haemorrhagic stroke in primary prevention?	In menopausal women we suggest against the use of HRT to reduce the risk of haemorrhagic stroke.	
	Quality of evidence: Low ⊕⊕	
	Strength of recommendation: Weak against intervention ↓	
1.3 In menopausal women with acute ischaemic stroke, does prior HRT compared with non-prior HRT impact functional outcome and mortality?	In menopausal women with acute ischaemic stroke we suggest against the use of HRT to reduce mortality. Quality of evidence: Very low ⊕	
	Strength of recommendation: Weak against intervention ↓	
2. Treatment of acute ischaemic stroke in pre-menopausal women (pregnancy, postpartum, and menstruation)	Since only data from case reports are available, a specific recommendation on IVT in pregnant women cannot be made.	A majority of members suggests that pregnant women with acute disabling ischaemic stroke, can be treated with IVT.
2.1 In pregnant women with acute ischaemic stroke does intravenous thrombolysis (IVT) improve outcome as compared to no IVT?		
2.2 In women with acute ischaemic stroke during the postpartum period does IVT improve outcome as compared to no IVT?	Since only data from case reports are available, a specific recommendation on IVT in postpartum women cannot be made.	All members suggest that postpartum women, occurring at least 10 days after delivery, can be treated with IVT.
2.3 In women with acute ischaemic stroke during menstruation does IVT improve outcome as compared to no IVT?	Since only data from case reports are available, a specific recommendation on IVT in women during menstruation cannot be made.	All members suggest that women with acute ischaemic stroke during menstruation, can be treated with IVT.
2.4 In women with acute ischaemic stroke during pregnancy does mechanical thrombectomy (MT) or intraarterial thrombolysis (IAT) improve outcome as compared to no MT and/or IVT?	Since only data from case reports are available, a specific recommendation on MT or IAT in pregnant women cannot be made.	All members suggest that pregnant women with stroke and large vessel occlusion can be treated with MT.
		A majority of members suggests that in pregnant women MT alone should be preferred over IVT or bridging therapy (IVT + ET).
2.5. In women with acute ischaemic stroke during postpartum period does endovascular treatment improve outcome as compared to no endovascular treatment and/or IVT?	No data, case reports available	It is reasonably plausible that postpartum women with stroke might benefit from MT.
		Furthermore, a majority of members suggests that is reasonably plausible to prefer MT alone over IVT or bridging therapy (IVT + ET)

A healthy baby was born to 28 patients; among patients treated with IVT alone, pregnancy was medically terminated (MTP) for two patients, one of them suffered an intrauterine haematoma, in one reason is unknown and in one case, MTP was requested. Among patients treated with IVT + MT, one had a miscarriage.

Intracerebral haemorrhage occurred in three patients (2 receiving single IVT, one treated with bridging therapy); all had a good outcome. One patient experienced intrauterine haematoma, which recovered.^25,41,53^ (Table 3, Supplementary Table 2)

**Evidence-based Recommendation**

Available data do not allow a specific recommendation on IVT in pregnant women with acute ischaemic stroke.

**Expert consensus statement**

A majority of members suggests that pregnant women with acute disabling ischaemic stroke, who otherwise meet eligibility criteria, can be treated with IVT after appropriately assessing the benefit/risk profile on an individual basis.

**Table 3.** Grade evidence profile table for PICO 2.1.

Certainty assessment							Impact	Certainty	Importance
№ of cases	Study design	Risk of bias	Inconsistency	Indirectness	Imprecision	Other considerations			
Maternal recovery									
33	Case report	Serious^a^	Not serious	Not serious	Not assessed	Publication bias strongly suspected^b^	Patients improved or had good recovery, 32 out of 33 cases (97%)	⊕○○○ VERY LOW	CRITICAL
Healthy baby									
32	Case report	Serious^a^	Not serious	Not serious	Not assessed	Publication bias strongly suspected^b^	Healthy baby 28 out of 32 cases, (87.5%)	⊕○○○ VERY LOW	CRITICAL
Abortion or medical termination of pregnancy									
32	Case report	serious^a^	not serious	not serious	not assessed	publication bias strongly suspected^b^	Abortion or MTP, 4 out of 32 cases (13%)	⊕○○○ VERY LOW	CRITICAL
Intracranial haemorrhage									
33	Case report	serious^a^	not serious	not serious	not assessed	publication bias strongly suspected^b^	Intracranial haemorrhage 3 out of 33 cases (9%)	⊕○○○ VERY LOW	CRITICAL
Intrauterine bleeding									
33	Case report	serious^a^	not serious	not serious	not assessed	publication bias strongly suspected^b^	Intrauterine bleeding, 1 out of 33 cases, (3%)	⊕○○○ VERY LOW	IMPORTANT

CI: Confidence interval.

^a^Not evaluated as these are case reports.

^b^Only a few case reports mentioned this outcome in one arm, MTP: medically terminated pregnancy.

### PICO 2.2. In women with acute ischaemic stroke during pregnancy, does mechanical thrombectomy (MT) or intraarterial thrombolysis (IAT) improve outcome compared to MT and/or IVT or IAT?

#### Analysis of current evidence

Currently, there are no RCTs on the use of acute stroke treatments with MT in pregnant women.

#### Additional information

Like alteplase treatment, only case reports have been published on MT or IAT of acute stroke in pregnant women. Of the 23 included case reports, 15 patients were treated with MT alone; 4 of them, treated before 2009, received just intraarterial alteplase (3 patients) or urokinase (1 patient). In 11 patients, different endovascular devices (Penumbra system, Solitaire AB stent, Stent retriever) were used. The remaining 8 patients were treated with a combination of IVT and MT (these subjects have also been taken into consideration for PICO 2.1.).^48–60^

All patients achieved a good outcome according to mRS (mRS 0–2) after 3 months; bleeding complications occurred in two patients only (a small intracerebral haemorrhage in the basal ganglia was observed in one patient receiving MT alone, and a haemorrhagic infarction type 1, that is, petechial haemorrhages at the infarct margins, occurred in one patient treated with bridging therapy); however, both had a favourable outcome. A healthy baby was born to 18 patients; one pregnancy ended in abortion, and in three cases, the birth and child data are missing. Another woman had MT after MTP, and a healthy baby was delivered. (Table 4, Supplementary Table 3)

**Evidence-based Recommendation**

Available data do not allow a specific recommendation on MT in women with acute ischaemic stroke during pregnancy.

**Expert Consensus Statement**

All members suggest that pregnant women with acute ischaemic stroke and large vessel occlusion, who otherwise meet eligibility criteria, can be treated with MT after appropriate assessment of the benefit/risk profile on an individual basis.

A majority of members suggests that in pregnant women with acute ischaemic stroke related to large vessel occlusion, and if MT is available, MT alone should be preferred over IVT or bridging therapy (IVT + MT).

**Table 4.** Grade evidence profile table for PICO 2.2.

Certainty assessment							Impact	Certainty	Importance
№ of cases	Study design	Risk of bias	Inconsistency	Indirectness	Imprecision	Other considerations			
Maternal recovery									
23	Case report	Serious^a^	Not serious	Not serious	Not assessed	Publication bias strongly suspected^b^	Maternal recovery was good to excellent in 23 out of 23 cases	⊕○○○ VERY LOW	CRITICAL
Healthy baby									
19	Case report	Serious^a^	Not serious	Not serious	Not assessed	Publication bias strongly suspected^b^	Healthy baby was delivered in 18 out of 19 cases, 95%	⊕○○○ VERY LOW	CRITICAL
Abortion or medical termination of pregnancy									
19	Case report	Serious^a^	Not serious	Not serious	Not assessed	Publication bias strongly suspected^b^	Abortion or MTP occurred in 1 out of 19 cases, 5%	⊕○○○ VERY LOW	CRITICAL
Intracranial haemorrhage									
23	Case report	Serious^a^	Not serious	Not serious	Not assessed	Publication bias strongly suspected^b^	Intracerebral haemorrhage occurred in 2 out of 23 cases ,9%	⊕○○○ VERY LOW	IMPORTANT
Intrauterine bleeding									
23	Case report	Serious^a^	Not serious	Not serious	Not assessed	Publication bias strongly suspected^b^	No case reported intrauterine bleeding	⊕○○○ VERY LOW	IMPORTANT

CI: Confidence interval.

^a^Not evaluated in these case reports.

^b^Only a few case reports mentioned this outcome, MTP:medically terminated pregnancy

### PICO 2.3. In women with acute ischaemic stroke during the postpartum period, does IVT improve outcome compared to no IVT?

#### Analysis of current evidence

Currently, there are no RCTs on the use of acute stroke treatments with IVT in the postpartum period.

#### Additional information

As for IVT during the postpartum period (defined as ≥ 10 days < 3 months after delivery) in the GWTG US Stroke Registry study, outcomes of pregnant women and women in the postpartum period treated with revascularization therapy were evaluated overall and not separately.^3^ The results are reported in question 2.1. We found only 2 published case reports about IVT in the postpartum period; one patient had a good outcome (mRS 0 after 6 weeks), and the second one clinical outcome was not reported. However, there were no bleeding complications in both patients. The first patient was treated 10 days after delivery while the second one 2 months later. Therefore, we do not have any currently available data-even from case reports–for less than 10 days.^61,62^ (Supplementary Table 4)

**Evidence-based Recommendation**

Available data do not allow a specific recommendation on IVT in postpartum women with acute ischaemic stroke.

**Expert Consensus Statement**

All members suggest that postpartum women with disabling ischaemic stroke, occurring at least 10 days after delivery, who otherwise meet eligibility criteria, can be treated with IVT with alteplase after appropriate assessment of the benefit/risk profile on an individual basis.

### PICO 2.4. In women with acute ischaemic stroke during the postpartum period, does MT or IAT improve outcome compared to no MT and/or IVT or IAT?

#### Analysis of current evidence

Currently there are no RCTs on women during the postpartum period receiving MT.

#### Additional information

For the postpartum period (as defined above) only five case reports were published in women treated with IAT from 1994 to 2010.^63–67^ All women were treated with IAT without MT, four of them reporting a good recovery while one, with basilar and internal carotid occlusion, died. There were no bleeding complications. (Supplementary Table 5)

**Evidence-based Recommendation**

Available data do not allow a specific recommendation on MT in women with acute ischaemic stroke during the postpartum period (defined as ≥ 10 days < 3 months).

**Expert Consensus Statement**

Although there are no currently available data waiting for evidence from clinical studies, it is reasonably plausible that postpartum women with acute ischaemic stroke, who otherwise meet eligibility criteria, might benefit from MT after appropriate assessment of the benefit/risk profile on an individual basis.

Furthermore, a majority of members suggests that, based on the time of stroke onset from delivery, if the risk of bleeding is deemed high, and if MT is available, it is reasonably plausible to prefer MT alone over IVT or bridging therapy (IVT + MT) on an individual basis.

### PICO 2.5. In women with acute ischaemic stroke during menstruation, does IVT improve outcome as compared to no IVT?

#### Analysis of current evidence

Currently, there are no RCTs on the use of IVT in women during menstruation.

#### Additional information

There is little literature regarding the safety of IVT during menstruation. Wein et al. described 5 subjects in the active arm of the National Institute of Neurological Disorders and Stroke (NINDS) IVT trial, which were coded as actively menstruating. Of these cases, only one, which was described in detail, required transfusion but had a good outcome in terms of NIHSS score after 3 weeks. Of the remaining four cases presented in summary, one subject with a 1-year history of dysfunctional uterine bleeding required urgent uterine artery ligation.^12^ The clinical case described by Chandran et al. did not have any complications and achieved a good outcome in terms of NIHSS score at discharge.^68^ (Supplementary Table 6)

**Evidence-based Recommendation**

Available data do not allow specific recommendation on IVT in women with ischaemic stroke during menstruation.

**Expert Consensus Statement**

All members suggest that women with acute ischaemic stroke during menstruation, who otherwise meet eligibility criteria, can be treated with IVT with alteplase after appropriate assessment of the benefit/risk profile on an individual basis.

A summary of recommendations is given in table 5. The results of the expert consensus member voting are shown in Supplementary Table 7.

## Discussion

This guideline document was developed following the GRADE methodology and aimed to assist physicians in decision-making regarding the risk of stroke in postmenopausal women related to HRT and the risk of stroke in pregnant/postpartum women treated by IVT and MT. Whenever possible, we based our recommendations on RCTs rather than observational studies, which are more prone to selection bias and confounding. Where insufficient scientific evidence was available, we provided expert consensus statements based on observational studies and our expertise. We found that there is low-quality evidence for recommendations on HRT in postmenopausal women. Based on the results of six RCTs,^18–23^ we suggest against HRT in postmenopausal women to reduce the risk of ischaemic or haemorrhagic stroke. Prior HRT has no impact on mortality in postmenopausal women with an acute stroke. However, there are limitations of the available RCT-based evidence. The currently used hormone replacement medication to control perimenopausal and postmenopausal symptoms has changed since the publication of trials results in the 1990ies. No trial was designed to investigate the risk or outcome of women with HRT, and stroke and stroke subtypes were not separately investigated. Moreover, no information on HRT in women with a previous stroke is available. The trial results on stroke mortality need to be interpreted with caution as acute stroke patient management and treatment have evolved dramatically over the last 2 decades, and today’s treatment cannot be compared to acute stroke treatment in the 1990s. The literature search did not identify trials that targeted postmenopausal women with acute ischaemic or haemorrhagic stroke with prior HRT therapy compared with non-prior HRT, impacting functional outcome and mortality.

We concluded to recommend against HRT as the overall results did not show a clear benefit or reduced risk in developing a stroke. The strengths of this guideline are its systematic approach to searching the literature and guidance by the GRADE recommendations. However, most RCTs included predominantly healthy postmenopausal women. The next priority of research related to sex-specific stroke management is to address the clinical outcome after an acute stroke of postmenopausal women treated with HRT and to focus on women to prevent stroke and improve recovery at the age around menopause, which should include women of different age groups, and with relevant comorbidities (e.g. autoimmune diseases) and/or vascular risk factors to allow subgroup analyses and improve and specify recommendations for HRT.

During pregnancy and in postpartum period, haemodynamic changes, the hypercoagulable state, hypertensive disorders of pregnancy and their complications contribute to the increased risk of stroke, on the other hand, acute treatment of stroke is associated with the risk of bleeding.^7,8^ Regarding stroke in pregnant women, a higher stroke risk and higher case fatality was observed.^69^ Not much is known about the toxicity and long-term effects of recombinant tissue plasminogen (rtPA) for the mother and foetus.

Pregnant women had been excluded from all RCTs, and therefore, we lack data on the effectiveness and safety of acute treatment in this group of patients. Based on observational data from the US GWTG Stroke Registry intravenous alteplase is listed with the U.S. Food and Drug Administration (FDA) as pregnancy category ‘C’ according to the package label, indicating ‘possible risk’ only.^3^

Following the low quality of data and the lack of RCTs, we included expert consensus statements in recommendations to guide clinicians in their everyday clinical practice. In this statement, a consensus was reached with a majority of members suggesting acute treatment with MT/IVT during pregnancy, postpartum and menstruation in patients who otherwise meet eligibility criteria after appropriate assessment of the benefit/risk profile on an individual basis. These recommendations are also in line with the results of a recent survey of the Canadian Stroke Consortium.^70^ Whenever possible, MT should be preferred over IVT. However, if MT is not accessible, IVT should not be withheld.

The risk and benefit to both mother and foetus should be considered when deciding to administer IVT. According to the Canadian Stroke best Practice Consensus Statement, ‘Acute stroke management during pregnancy’, maternal health is prioritized, and delays or deferral of critical steps in diagnosis and lifesaving care due to pregnancy should be minimized.^7^ Despite short time for decision management of acute stroke in pregnant women multiple specialities have to be involved including advanced obstetric care. This includes transfer to a hospital with appropriate neurological and obstetrical expertise, and, if this is not possible, telemedicine should be used*.*

We found a limited series of case reports (approximately 33: 25 with IVT alone and 8 combinations of IVT and MT in April 2021). According to these, the use of thrombolytics may be feasible in pregnant patients in all trimesters, with the benefits of IVT outweighing the risks. Most of the patients received rtPA with a dosage of 0.9 mg/kg. It is not reported whether the weight on which the rtPA dosage was based on the actual body weight during pregnancy or not. According to Ryman et al the dose should reflect the patient’s current body weight and do not support a dose adjustment for a patient´s non-pregnant weight.^43^

Similar to IVT, also for MT or intraarterial thrombolysis in pregnant women (23 case reports), maternal recovery was good to excellent in all patients. We consider MT as safe and effective for acute stroke in patients with large vessel occlusion, which is consistent with the conclusion of Dicpinigaitis et al.^71^ However, our conclusions are based on case studies that had no uniform assessment of outcome.

The lack of strong evidence regarding the stroke treatment of pregnant women is widely regarded as unfair.^72^ In 1993, the Council for International Organizations of Medical Sciences claimed that the exclusion of pregnant women from clinical trials as a class is unjust.^73,74^ The view that pregnant women should be enrolled to clinical research was later supported by regulatory agencies (US Food and Drug Administration^75^ and the European Medicines Agency).^76^ Despite this longstanding consensus on the need to include pregnant women in clinical research, the situation has not significantly changed since 1994. Still, this position is untenable, as it leaves physicians and patients with inadequate data on which to base prescribing decisions for pregnant women.^77,78^

Fair inclusion of pregnant women means 1. that pregnant women who are eligible are not excluded solely for being pregnant and 2. that the research interests of pregnant women are prioritized, meaning that they ought to receive substantially more attention.

Accordingly, for a better evaluation of the management of pregnant/postpartum women with acute stroke, the SiPP (Stroke in Pregnancy and Postpartum), a prospective, observational, international, multicentre study on pathophysiological mechanisms, clinical profile, management and outcome of cerebrovascular diseases in pregnant and postpartum women was started.^79^

## Plain language summary

In this guideline document, we focused on two substantial phases in female lives, in which vulnerability is high, and stroke risk and treatment need adjustment. It is debatable whether postmenopausal women treated with HRT have an increased risk for ischaemic or haemorrhagic stroke. Also, acute stroke treatment of pregnant, postpartum or menstruating women is considered risky, and often treatment is withheld due to a lack of available guidance.

This guideline document addresses both questions and offers expert guidance based on a systematic review and meta-analysis of the current literature. Where evidence creating results lacked, the provided guidance was based on the expert opinion of the involved working group members. The GRADE methodology was applied to develop this guideline. Based on the results from 6 randomized controlled clinical trials, which provide the highest evidence available to answer a research question, we gave a weak recommendation against the use of HRT in postmenopausal women. This was based on the fact that no reduction in the overall risk for a stroke or the mortality rate was found. The recommendation is limited by the fact that most trials were performed >20 years ago, women participating in these trials were mostly healthy individuals, and trials were not designed to understand outcome after ischaemic or haemorrhagic stroke.

The evidence available to guide on acute stroke treatment in pregnant or menstruating women or women after having given birth is even more limited. No RCTs are available and even further most trials investigating acute stroke treatment excluded these groups of participants. Therefore, no recommendation based on evidence data could be given. However, in expert consensus statements the working group members favoured the treatment of pregnant or menstruating women or women after having given birth with the intravenous clot buster medication – thrombolysis – and/or the interventional brain catheter treatment – mechanical thrombectomy – in case of acute ischaemic stroke, in which these treatments would be indicated.

The working group has concluded that further research is needed to increase the limited data available.

## Supplemental Material

sj-pdf-1-eso-10.1177_23969873221078696 – Supplemental Material for European Stroke Organisation guidelines on stroke in women: Management of menopause, pregnancy and postpartum Click here for additional data file. Supplemental Material, sj-pdf-1-eso-10.1177_23969873221078696 for European Stroke Organisation guidelines on stroke in women: Management of menopause, pregnancy and postpartum by Christine Kremer, Zuzana Gdovinova, Yannick Bejot, Mirjam R. Heldner, Susanna Zuurbier, Silke Walter, Avtar Lal, Corina Epple, Svetlana Lorenz, Marie-Luise Mono, Theodore Karapanayiotides, Kailash Krishnan, Dejana Jovanovic, Jesse Dawson, Valeria Caso in European Stroke Journal

sj-pdf-2-eso-10.1177_23969873221078696 – Supplemental Material for European Stroke Organisation guidelines on stroke in women: Management of menopause, pregnancy and postpartum Click here for additional data file. Supplemental Material, sj-pdf-2-eso-10.1177_23969873221078696 for European Stroke Organisation guidelines on stroke in women: Management of menopause, pregnancy and postpartum by Christine Kremer, Zuzana Gdovinova, Yannick Bejot, Mirjam R. Heldner, Susanna Zuurbier, Silke Walter, Avtar Lal, Corina Epple, Svetlana Lorenz, Marie-Luise Mono, Theodore Karapanayiotides, Kailash Krishnan, Dejana Jovanovic, Jesse Dawson, Valeria Caso in European Stroke Journal
