# Supplementary material for: European Stroke Organisation guidelines on stroke in women: Management of menopause, pregnancy and postpartum
Source: Eur Stroke J. 2022 Mar 29;7(2):I–XIX. doi: 10.1177/23969873221078696 (PMC9134774; doi:10.1177/23969873221078696)
Supplement: sj-pdf-1-eso-10.1177_23969873221078696 – Supplemental Material for European Stroke Organisation guidelines on stroke in women: Management of menopause, pregnancy and postpartum [file sj-pdf-1-eso-10.1177_23969873221078696.pdf]

Table 1: Selected outcomes for each PICO and respective average voting for importance of the members

PICO 1		PICO 2	
Outcomes	Average	Outcomes	Average
Stroke, all	9	mRS at 90 days	9
Stroke, ischaemic	9	recanalization (MT)	7
Stroke, haemorrhagic	9	Treatment complications (MT)	7
CVT	8	Early neurological improvement	6
Transient Ischaemic attack	8	Mortality, all cause, overall	9
Myocardial infarction (MI)	6	Intra-hospital mortality	9
Cardiovascular and MI events	7	Quality of life	5
Angina	6	Haemorrhage	9
Coronary disease	6	Major bleeding	9
Sytemic embolism	7	Minor bleeding	6
Mortality, all cause	9	Intracerebral haemorrhage	9
Mortality, cardiac	6	Symptomatic cerebral haemorrhage	9
IMT	5	Intracranial bleeding	9
Functional outcome mRS 3 months	8	Gastrointestinal bleeding, major/minor	5
Depression	6	Gastrointestinal bleeding, major	9
Cognition/Dementia	6	Gastrointestinal bleeding, minor	6
Fatigue	6	Complication in pregnancy	9
Breast Cancer	6	Premature delivery	8

Quality of life, ADL	5	Abortion	9
Osteoporosis	5	Healthy baby	8
Fractures	6	Bleeding vaginal, major/minor	6
Major bleeding	7	Bleeding vaginal, major	6
Minor bleeding	6	Bleeding vaginal, minor	5
Bleeding vaginal, major	6	Bleeding placenta	6
Bleeding vaginal, minor	5		
Menopausal hot flushes	6		

CVT: cerebral venous thrombosis, IMT: Intima Media Thickness

Rating of outcomes points according to importance: 9-7 critical, 4-6 important, 0-3 less important

Table 2: Summary of case reports of intravenous thrombolysis alone or in combination with mechanical thrombectomy in pregnant women with acute ischaemic stroke

**Authors**	**Age; Gestation**	**Treatment (route; dose)**	**Stroke description**	**NIHSS score**	**Haemorrhage**	**Clinical Outcome**	**Fetal outcome**
Dapprich et al, 2002	31 years; 12 weeks	IV rt-PA; 0.9mg/kg	Right - sided hemiplegia and global aphasia. L-MCA occlusion; protein S deficiency.	NR	Small Left basal ganglia haemorrhagic infarction	Improved. L-MCA reopened.	Healthy baby
Leonhardt et al, 2006	26 years; 23 weeks	IV rt-PA; 0.9mg/kg	R-sided hemiparesis. L-MCA occlusion. anti-phospholipid antibodies; protein S deficiency.	NR	No	Good improvement. L-MCA partial reocclusion.	Healthy baby
Murugappan et al, 2006	37 years; 12 weeks	IV rt-PA; 0.9mg/kg	Left-sided weakness and numbness, lower facial droop, dysarthric speech. R-MCA occlusion; mitral valve replacement embolism.	19	Intraut. haematoma	Recovered well.	MTP
Murugappan et al, 2006	31 years; 12 weeks	IV rt-PA; 0.9mg/kg	Right-sided hemiparesis and severe expressive aphasia. L-MCA occlusion; protein S deficiency.	19	No	Improvement in right-sided motor function and speech. NIHSS score - 4 after 4 weeks.	MTP
Wiese et al, 2006	33 years; 13 weeks	IV rt-PA; 0.9mg/kg	Right-sided hemiparesis and expressive aphasia. L-MCA occlusion.	13	No	Good improvement. NIHSS score -	Healthy baby CS

Authors	Age; Gestation	Treatment (route; dose)	Stroke description	NIHSS score	Haemorrhage	Clinical Outcome	Fetal outcome
			mitral valve replacement.			11, then 4.	
Yamaguchi et al, 2010	36 years; 18 weeks	IV rt-PA; 0.6mg/kg	Right-sided hemiparesis, motor aphasia. L-MCA occlusion; Factor VIII elevation (>200%); protein C resistance.	6	No	Recovered Well.	Healthy baby
Ratajczak et al, 2012	33 years; 6 weeks	IV rt-PA; Standard dose	Right-sided hemiparesis and expressive aphasia. Restricted diffusion in three different areas in the left hemisphere; PFO and right-left shunt.	NR	No	Neurological symptoms improved rapidly.	Healthy baby
Hori et al, 2013	35 years; 14 weeks	IV rt-PA; 0.6mg/kg	Left -sided visual field defect, hemiparesis, and dysesthesia. R-PCA occlusion. 35% patients - protein S deficiency.	NR	No	Improved	Healthy baby CS
Karunaratne et al, 2013	30 years; 38 weeks	IV tPA; Dose NR	Left-sided weakness.	12	NR	Symptoms improved rapidly. NIHSS score - 1 after 2 days	Healthy baby
Tassi et al, 2013	28 years; 16 weeks	IV rt-PA; 0.9mg/kg	Right-sided hemiparesis and hypoesthesia, motor aphasia, L-MCA subocclusion; paradoxical embolism due to PFO and Factor V Leiden mutation.	20	No	Motor aphasia, hemiparesis improved within a few hours. NIHSS score-1 after 1 day.	Healthy baby
Mantoan Ritter L et al, 2014	32 years; 16 weeks	IV rt-PA; 0.9mg/kg	Left middle cerebral artery syndrome - dysphasia, dysarthria, right-sided hemianopia, hemiplegia, and hemisensory loss. L-MCA M2 segment occlusion	22	No	NIHSS score – 13 4 month later – mild residual hemiparesis, fully independent mRS-2	Healthy baby CS
Ritchie et al, 2015	28 years; 39 weeks	IV rt-PA; NR	Left-sided hemiparesis, facial weakness and tongue deviation, impaired sensation. R-MCA occlusion. Cryptogenic stroke.	11	No	Fully recovered. NIHSS score - 6 after 1 day	Healthy baby, forceps assisted vaginal delivery
Tversky et al, 2016	31 years; 5 weeks	IV t-PA; NR	Right-sided mild hemiparesis, slurred speech, hemisensory loss. L-MCA occlusion; paradoxical embolism due to PFO; protein C and S deficiency.	5	No	Full recovery	Healthy baby
Reining-Festa et al, 2017	37 years; 5 weeks	IV rt-PA; Standard dose	Left-sided hemiplegia. Restricted diffusion of R-MCA.	8	No	Recovered well. NIHSS score-4, mRS after 3 months – 1.	Healthy baby, CS
Kalcik M et	28 years;	t-PA 25	Thrombus on the mitral	NA	No	Hemiplegia	Healthy

Authors	Age; Gestation	Treatment (route; dose)	Stroke description	NIHSS score	Haemorrhage	Clinical Outcome	Fetal outcome
al, 2017	24 weeks	mg/2h	prosthesis<br>Right hemiplegia<br>Thrombotic occlusion of left MCA			resolved	baby was delivered
Khan A et al., 2017	33 years; 9 weeks	NR	Right-sided hemiparesis, hemisensory loss, dysarthria and homonymous hemianopia<br>(History - eleven miscarriages of, cocaine, heroin, cannabis abuse)	NA	No	Mild fine motor incoordination in the right hand, mild dysarthria, and right homonymous hemianopia	At day three postthrombolysis the patient requested a termination of pregnancy
Landais et al, 2018	32 years; 13 week	IV rt-PA; 0.9mg/kg	Right hand numbness and aphasia.<br>M2 segment L-MCA occlusion.		No	Recovered Well.	Healthy baby
Jiang Z and Hu Z, 2018	26 years; 31 week	IV rt-PA; Standard dose	Right sided hemiparesis, slurred speech	6	Asympt. haemorrhage in left cerebellar hemisphere and right cortex	NIHSS score 3, after haemorrhage 4 (mitral valve prolapse)	Healthy baby, Vaginal delivery
Shah SS et al, 2018	37 years; 9 weeks	IV rt-PA; 0.9mg/kg<br><br>MT	Acute left-sided hemiplegia and right gaze preference M2 segment R-MCA occlusion.<br><br>2 days later – again left-sided hemiplegia and right gaze preference	9<br><br>13		NIHSS score 4, Left-sided hemiplegia and right gaze preference resolved<br>EVT – recanalization TICI 2b<br>NIHSS - 3	
Ryman KM et al, 2019	26-years 12 weeks	IV rt-PA; 0.9mg/kg	Expressive aphasia and right hemiparesis, after 90 min resolution of symptoms<br>30 min later less severe symptoms	29<br><br>4	No	Without residual deficits	Unspecified complications of prematurity. No birth defects Delivery without complications
Ryman KM et al, 2019	24 years, 30 weeks	IV – rtPA – dose NR	Right hemiparesis, right upper extremity sensory loss, and dysarthria	12	No	Marked neurologic improvement	Healthy baby at Without complications
Peksa GD et al, 2019	35 years, 9 weeks	IV – rtPA 0.9 mg/kg	Lost balance and unable to move the left side of body<br>Right M1-MCA filling defect (CT AG)	7	No	NIHSS 0, mRS 1	Healthy baby (hyperbilirubinemia) vaginal delivery
Rodrigues R et al, 2019	29 years 27 weeks	IV – rtPA	Aphasia, right-sided hemiplegia, hemianopsia	23	No	Motor aphasia and right	Healthy baby

Authors	Age; Gestation	Treatment (route; dose)	Stroke description	NIHSS score	Haemorrhage	Clinical Outcome	Fetal outcome
			Occlusion of the proximal left MCA			hemiparesis persisted, gait with bilateral support, NIHSS 14.	Cesarean delivery
Aaron S et al, 2020	35 years; 39 weeks	IV – rtPA 0.9 mg/kg	Right-sided weakness and inability to speak	11	No	*Improvement in right hemiparesis improved fully and language*<br><br>*At 3 months, word finding difficulty for low frequency words*	Healthy baby, Vaginal delivery
Bojda M et al, 2021	31 years, 34 weeks	IV – rtPA 0.9 mg/kg	Slurred speech, severe right hemiparesis, facial palsy, psychomotor agitation	16	No	NIHSS 4 after 7h, 1 after 10 days	Healthy male infant Vaginal delivery
Bhogal P et al, 2017	36 years, 25 weeks	36 mg IV rt-Pa, combined with MT with aspiration catheter and later MT with stent retriever	Headache, blurred vision, and nausea, several episodes of vomiting, than rapid deterioration of consciousness and patient became stuporous.<br>Distal occlusion of the basilar artery.	NR	No	mRS - 1	CS, healthy baby
Zhu F et al, 2018	28 years, 9 weeks	0.9mg/kg IV rt-PA combined with MT with aspiration	Right motor and sensitive deficit with dysarthria experienced on waking.<br>M1 segment L- MCA occlusion.	13	No	NIHSS score - 1	Healthy baby, vaginal delivery
Watanabe TT et al., 2019	36 years 21 weeks	IV rtPA 0.6mg/kg Penumbra, Trevo XP	Dysarthria and right hemiparesis<br>Left ICA occlusion	13	No	NIHSS score – 4,<br>mRS 1 after delivery	Healthy baby, Delivery without complications
Kristiansen E et al., 2019	26 years 33 weeks	IV rtPA 90 mg<br>MT - Trevo stent retriever	Left-sided paralysis, facial weakness, inattention, dysarthria, and incomplete left vertical gaze palsy<br>Right M1- MCA occlusion	14	No	2 weeks after disacharge - slight facial palsy, reduced fine motor skills of the left arm, and slight cognitive deficits	Healthy baby
Tse GH et al., 2019	36 years, 8 weeks	IV tPA<br>Stent-retriever mechanical thrombectomy	Dysphasia and right hemiplegia<br>Thrombus in the proximal left MCA	21	No	Excellent recovery from hemiplegia, but dysphasia was slow to improve	Healthy baby<br><br>Uneventful delivery
Limaye K et	NA	IV tPA	Right M1-MCA occlusion	15	No	Discharge	Normal full-

Authors	Age; Gestation	Treatment (route; dose)	Stroke description	NIHSS score	Haemorrhage	Clinical Outcome	Fetal outcome
al., 2020	Second trimester	SMAT				NIHSS – 3<br>90 days mRS - 1	term delivery
Limaye K et al., 2020	NA<br>First trimester	IV tPA<br>ADAPT	Right M1-MCA occlusion	11	HI-1	Discharge NIHSS – 1<br>90 days mRS - 1	Spontaneous abortion
Limaye K et al., 2020	NA<br>Third trimester	IV tPA<br>SMAT using BGC	Right M1-MCA occlusion	12	No	Discharge NIHSS – 1<br>90 days mRS - 1	Normal full-term delivery

ADAPT – a direct aspiration first pass technique; BGC – balloon guide catheter; CT AG - Computed tomography angiography, CS – Cesarean section; HI-1 – haemorrhagic infarction type 1; ICA – internal carotid artery; IV: Intravenous; IVT – intravenous thrombolysis, L-MCA: Left middle cerebral artery; mRS: modified Rankin Scale score; MAT – mechanical aspiration thrombectomy; MT – mechanical thrombectomy; MTP: Medical termination of pregnancy; NIHSS: National Institutes of Health Stroke Scale; NR: Not reported; ; NA – Not available; PFO: Patent foramen ovale; R-MCA: Right middle cerebral artery; rt-PA: Recombinant tissue plasminogen activator; R-PCA: Right posterior cerebral artery; SMAT – stent assisted mechanical aspiration thrombectomy; tPA: Tissue plasminogen activator; TE – thrombectomy;

Table 3: Summary of case reports of mechanical thrombectomy and intraarterial thrombolysis in pregnant women with acute ischaemic stroke

**Authors**	**Age/gestation**	**Treatment route; dose**	**Stroke description**	**NIHSS score**	**Haemorrhage**	**Outcome**	**Fetal outcome**
Elford et al, 2002	28 years, 7 days after transfer of embryo	IA 15.5 mg rtPA	Left hemiplegia, dysarthria, left facial paralysis, and drowsiness. M1 segment R-MCA occlusion.	11	Small 1.5- 2.0 cm hematoma in the right basal ganglia, later to 3 cm	NIHSS score 3, Deterioration, at follow-up, mRS 1-2	Healthy male infant, vaginal delivery
Denschlag et al, 2005	28 years, 25 weeks	IA 30mg rtPA	Eye deviation, horizontal nystagmus, paralysis of the upper extremities and dysarthria. BA thrombosis	NR	No	Good outcome. Complete resolution of the mitral thrombus under warfarin therapy.	Pregnancy terminated by combined CS and HE because of mitral valve thrombus<br><br>Thriving infant
Johnson DM et al, 2005	39 years, 37 weeks	IA 15 mg rtPA	Left-sided hemiplegia, decreased responsiveness. M1 segment R-MCA occlusion.	20	No	NIHSS score 7 after 9 hours	Healthy baby, forceps assisted vaginal delivery
Yamada N et al, 2010	34 years, 39 week	IA urokinase	Left side semiparalysis and articulation disorder.	8	No	Recovered well	NR
Aaron S et al, 2016	24 years, 3rd trimester	MT with Penumbra system	Left-sided hemiplegia, hemineglect and altered sensorium. M1 segment R-MCA occlusion.	20	No	NIHSS score 12, mRS – 1 at discharge, after 6 months - 0	Healthy baby, vaginal delivery
Aaron S et al, 2016	28 years, 37 weeks	MT with Penumbra system	Left hemiplegia and Drowsiness. M1 segment R-MCA occlusion.	21	No	NIHSS score 4, mRS after 6 months - 2	Healthy baby girl, vaginal delivery
Bhogal P et al, 2017	38 years, 24 weeks	Solitaire AB Stent, manual aspiration, IA 9 mg rtPA	Right-sided hemiplegia and hemianaesthesia, aphasia, deviated gaze to the left. Terminal internal carotid artery (ICA) occlusion.	15	No	mRS - 1	Healthy baby, vaginal delivery
Blythe R et al, 2019	29 years, 39 weeks	Clot aspiration	Left facial paresis, hemiparesis, and neglect Large occlusive thrombus within the right M1- M2 segments	11	No	mRS 0 after 6 weeks	Caesarean section 5 days after MT, delivering a healthy new born
Tse GH et al., 2019	28 years, 39 weeks	Thrombus aspiration with Penumbra	Right hemiplegia, facial droop and dysarthria Left M1-MCA occlusion	11	No	Minimal residual facial weakness	Elective caesarean section at 40 weeks with safe delivery of

		system					child
Tse GH et al., 2019	27 years, 36 weeks	MT - stent retriever	Right-sided hemiplegia Proximal left MCA thrombus, complete occlusion, good collaterals	22	No	No significant neurological deficit	Normal delivery
Limaye K et al., 2020	NA Second trimester	SMAT using BGC	Left M1-MCA occlusion	18	No	Discharge NIHSS – 5 90 days mRS - 2	NA
Limaye K et al., 2020	NA Third trimester	3xMAT, 2xstent retriever, rescue stenting	Right ICA terminus occlusion	12	No	Discharge NIHSS – 0 90 days mRS - 0	Normal full-term delivery
Limaye K et al., 2020	NA Third trimester	MAT	Right M1-MCA occlusion	28	No	Discharge NIHSS – 0 90 days mRS - 0	Normal full-term delivery
Limaye K et al., 2020	NA First trimester	SMAT	Left M1-MCA occlusion	9	No	Discharge NIHSS – 2 30 days mRS - 0	NA
Wiacek M et al., 2020	27 years, 35 week	Mechanical thrombectomy	dysarthria, motor aphasia, partial right-sided hemianopia and hemiparesis Cesarean section, then DSA - left MCA occlusion	15	No	24 hours NIHSS – 7 Discharge NIHSS – 1 After 3 months NIHSS – 0, mRS - 0	Healthy baby, Cesarean section before MT
Bhogal P et al, 2017	36 years, 25 weeks	36 mg IV rt-Pa, combined with MT with aspiration catheter and later MT with stent retriever	Headache, blurred vision, and nausea, several episodes of vomiting, than rapid deterioration of consciousness and patient became stuporous. Distal occlusion of the basilar artery.	NR	No	mRS - 1	CS, healthy baby
Zhu F et al, 2018	28 years, 9 weeks	0.9mg/kg IV rt-PA combined with MT with aspiration	Right motor and sensitive deficit with dysarthria experienced on waking. M1 segment L- MCA occlusion.	13	No	NIHSS score - 1	Healthy baby, vaginal delivery
Watanabe TT et al., 2019	36 years 21 weeks	IV rtPA 0.6mg/kg Penumbra, Trevo XP	Dysarthria and right hemiparesis Left ICA occlusion	13	No	NIHSS score – 4, mRS 1 after delivery	Healthy baby, Delivery without complications
Kristiansen E et al., 2019	26 years 33 weeks	IV rtPA 90 mg MT - Trevo stent retriever	Left-sided paralysis, facial weakness, inattention, dysarthria, and incomplete left vertical gaze palsy Right M1- MCA occlusion	14	No	2 weeks after disacharge - slight facial palsy, reduced fine motor skills of the left arm, and slight cognitive	Healthy baby

						deficits	
Tse GH et al.,<br>2019	36 years,<br>8 weeks	IV tPA<br>Stent-retriever mechanical thrombectomy	Dysphasia and right hemiplegia<br>Thrombus in the proximal left MCA	21	No	Excellent recovery from hemiplegia, but dysphasia was slow to improve	Healthy baby<br><br>Uneventful delivery
Limaye K et al.,<br>2020	NA<br>Second trimester	IV tPA<br>SMAT	Right M1-MCA occlusion	15	No	Discharge NIHSS – 3<br>90 days mRS - 1	Normal full-term delivery
Limaye K et al.,<br>2020	NA<br>First trimester	IV tPA<br>ADAPT	Right M1-MCA occlusion	11	HI-1	Discharge NIHSS – 1<br>90 days mRS - 1	Spontaneous abortion
Limaye K et al.,<br>2020	NA<br>Third trimester	IV tPA<br>SMAT using BGC	Right M1-MCA occlusion	12	No	Discharge NIHSS – 1<br>90 days mRS - 1	Normal full-term delivery

ADAPT – a direct aspiration first pass technique; BGC – balloon guide catheter; CS-Cesarean section; HE-Hysterectomy; HI-1 – haemorrhagic infarction type 1; IA: Intraarterial; IV: Intravenous; L-MCA: Left middle cerebral artery; mRS: modified Rankin Scale score; MAT – mechanical aspiration thrombectomy; NA – Not available; NIHSS: National Institutes of Health Stroke Scale; NR: Not reported; R-MCA: Right middle cerebral artery; rt-PA: Recombinant tissue plasminogen activator; MT – mechanical thrombectomy, ICA – internal carotid artery, SMAT – stent assisted mechanical aspiration thrombectomy, BGC – balloon guide catheter,

Table 4: Summary of case reports intravenous thrombolysis and mechanical thrombectomy in postpartum women with acute ischaemic stroke

**Authors**	**Age/ postpartum time**	**Treatment (route; dose)**	**Stroke description**	**NIHSS score**	**Haemorrhage**	**Clinical Outcome**
Bereczki et al, 2016	40 years; 10 days	IV rt-PA; 0.9mg/kg dissection of R-ICA - fixed by carotid Wallstent	Right-sided hemiparesis, central facial palsy, aphasia, conjugated gaze deviation to the left, anisocoria M1 segment L-MCA occlusion, R-ICA 65-70% stenosis, L-ICA occlusion	23	No	mRS – 1, after 6 weeks mRS - 0
Nasa P et al., 2021	39 years 2 months after delivery	IV rt-PA; dose – NR Enoxaparin, warfarin (peripartum cardiomyopathy (PPCM)	Left-sided numbness and weakness, facial asymmetry, and difficulty in speech	8	No	NR

ICA – internal carotid artery; IV: Intravenous; IVT – intravenous thrombolysis, L-MCA: Left middle cerebral artery; L-ICA: Left internal carotid artery; mRS: modified Rankin Scale score; R-ICA: Right internal carotid artery; rt-PA: Recombinant tissue plasminogen activator;

Table 5: Summary of case reports of intraarterial thrombolysis during the postpartum period

**Authors**	**Age/ postpartum time**	**Treatment route; dose**	**Stroke description**	**NIHSS score**	**Haemorrhage**	**Outcome**
Cincotta et al, 1995	22 years,<br>14 days after delivery	1,000,000 U of urokinase injected in increments of 200,000 U	Drowsy, but able to follow commands. Bilateral horizontal gaze palsies. Absent gag reflex, incomplete quadriparesis. Patient was on ventilation.<br>Basilar artery thrombosis.	NR	No	Recovery after 2 months, complete after 1 year
DeKoninck PLJ et al, 2008	33 years,<br>19 days after delivery	IA thrombolysis, dose NR	Dizziness, nausea, vomitus, than apnea and cardiac arrest.<br>Basilar and internal carotid occlusion.	NR	No	Death
Mendez JC et al, 2008	37 years,<br>15 hours after Cesarean delivery	IA urokinase 100,000 units over a period of 15 min.	Sudden onset of left face, arm, and leg hemiplegia, homonymous hemianopsia, and slight dysarthria.	16	No	NIHSS score -2, after 3 months without deficit
Tomita T et al, 2010	29 years,<br>3 days after delivery	IA 20 mg rt-PA	Right-sided hemiplegia, global aphasia.<br>M2 segment L-MCA occlusion.	14	No	Recovery, NIHSS score - 1 after 4 months
Ronning OM et al., 2010	29 years<br>3 days after delivery	Intra-arterial rt-PA – 20 mg	Tachycardia and dyspnoea with orthopnoea, a left ventricular thrombus was detected, then right-sided hemiplegia with global aphasia.<br>Left M2-MCA occlusion	14	No	Mild right facial paralysis, reduced tempo of the right hand, a partial non-fluent aphasia<br>After 4 months – NIHSS 1, mRS 1

IA: Intraarterial; L-MCA: Left middle cerebral artery; mRS: modified Rankin Scale score; NIHSS: National Institutes of Health Stroke Scale; NR: Not reported; rt-PA: Recombinant tissue plasminogen activator

Table 6: Summary of case reports of intravenous thrombolysis in women with acute ischaemic stroke during menstruation

**Authors**	**Menstruation**	**Treatment (route; dose)**	**Stroke description**	**NIHSS score**	**Haemorrhage**	**Clinical Outcome**
Wein TH et al, 2002	40 years; 20 hours of her menses.	IV rt-PA; 0.9mg/kg per NINDS protocol	Right-sided hemiparesis, dysphasia, dysarthria. M1 segment L-MCA occlusion	10	After 25 min. of IV rt-PA - marked increase in menstrual flow. IV fluids without response. Transfusion of 2 U of packed red blood cells.	NIHSS score – 5 after 3-weeks.
Chandran, 2015	36 years;	IV rt-PA; 0.9mg/kg per NINDS protocol	Left-sided hemiplegia, dysarthria. Occlusion M1 segment R-MCA.	12	No increase in her menstrual bleeding or drop in hemoglobin.	NIHSS score – 4 at discharge after 3-days.

IV: Intravenous; IVT – intravenous thrombolysis, L-MCA: Left middle cerebral artery; mRS: modified Rankin Scale score; NIHSS: National Institutes of Health Stroke Scale; R-MCA: Right middle cerebral artery; rt-PA: Recombinant tissue plasminogen activator; rt-PA: recombinant tissue plasminogen activator

Table 7: Synoptic table of the recommendations including all member votes of the expert consensus voting

PICO	**Recommendation**	**Expert consensus statement**
1. Hormone replacement therapy (HRT) and stroke risk<br>1.1. In menopausal women, does HRT compared to non-prior HRT reduce the risk of ischaemic stroke?	In menopausal women we suggest against the use of HRT to reduce the risk of ischaemic stroke.<br>Quality of evidence: Very low ⊕<br>Strength of recommendation: Weak against intervention ↓	
1.2 In menopausal women, does HRT compared to non-prior HRT reduce the risk of haemorrhagic stroke in primary prevention?	In menopausal women we suggest against the use of HRT to reduce the risk of haemorrhagic stroke.<br>Quality of evidence: Low ⊕⊕<br>Strength of recommendation: Weak against intervention ↓	
1.3 In menopausal women with acute ischaemic stroke, does prior HRT compared with non- prior HRT impact functional outcome and mortality?	In menopausal women with acute ischaemic stroke we suggest against the use of HRT to reduce mortality.<br>Quality of evidence: Very low ⊕<br>Strength of recommendation: Weak against intervention ↓	
2. Treatment of acute ischaemic stroke in pre-menopausal women (pregnancy, postpartum, and menstruation)<br>2.1 In pregnant women with acute ischaemic stroke does intravenous thrombolysis (IVT) improve outcome as compared to no IVT?	Since only data from case reports are available, a specific recommendation on IVT in pregnant women cannot be made.	A majority of members (12/13) suggests that pregnant women with acute disabling ischaemic stroke, can be treated with IVT.
2.2 In women with acute ischaemic stroke during the postpartum period does IVT improve outcome as compared to no IVT?	Since only data from case reports are available, a specific recommendation on IVT in postpartum women cannot be made.	All members (13/13) suggest that postpartum women, occurring at least 10 days after delivery, can be treated with IVT.
2.3 In women with acute ischaemic stroke during menstruation does IVT improve outcome as compared to no IVT?	Since only data from case reports are available, a specific recommendation on IVT in women during	All members (13/13)suggest that women with acute ischaemic stroke during

	menstruation cannot be made.	menstruation, can be treated with IVT.
2.4 In women with acute ischaemic stroke during pregnancy does mechanical thrombectomy (MT) or intraarterial thrombolysis (IAT) improve outcome as compared to no mechanical thrombectomy and/or IVT?	Since only data from case reports are available, a specific recommendation on MT or IAT in pregnant women cannot be made.	All members (13/13) suggest that pregnant women with stroke and large vessel occlusion can be treated with MT.<br>A majority (12/13) of members suggests that in pregnant women MT alone should be preferred over IVT or bridging therapy (IVT+ET).
2.5. In women with acute ischaemic stroke during postpartum period does endovascular treatment improve outcome as compared to no endovascular treatment and/or IVT?	No data, case reports available	It is reasonably plausible that postpartum women with stroke might benefit from MT . Furthermore, a majority of members (12/13) suggests that is reasonably plausible to prefer MT alone over IVT or bridging therapy (IVT+ET)
