# Supplementary material for: European Stroke Organisation guidelines on stroke in women: Management of menopause, pregnancy and postpartum
Source: Eur Stroke J. 2022 Mar 29;7(2):I–XIX. doi: 10.1177/23969873221078696 (PMC9134774; doi:10.1177/23969873221078696)
Supplement: sj-pdf-2-eso-10.1177_23969873221078696 – Supplemental Material for European Stroke Organisation guidelines on stroke in women: Management of menopause, pregnancy and postpartum [file sj-pdf-2-eso-10.1177_23969873221078696.pdf]

Declarations

I. Conflicts of interest of the group members:

Christine Kremer: Speaker fees: Boehringer Ingelheim, Bayer

Zuzana Gdovinova: Speaker fees: Boehringer-Ingelheim, MSD, Bayer, Novartis, Pfizer, Sandoz, TEVA. Advisory board member: Biogen, Boehringer-Ingelheim, Novartis, Pfizer, Shire, TEVA

Yannick Bejot: Speaker fees: BMS, Pfizer, Medtronic, NovoNordisk, Amgen, Servier, Boehringer-Ingelheim

Mirjam Heldner. No disclosures

Susanna Zuurbier: no disclosures

Avtar Lal: no disclosures

Corina Epple: Speaker fees: Portola

Svetlana Lorenzano: Consultant: Boehringer Ingelheim (2013-2014). Travel grants for conferences and meetings: Boehringer Ingelheim, Bayer, Quintiles IMS, DaichiiSankyo (one each).

Marie-Luise Mono: no disclosures

Theodore Karapanayiotides: Speaker fees: Boehringer-Ingelheim, Bayer, Pfizer, TEVA. Advisory Board member: Boehringer-Ingelheim, Novartis, TEVA

Kailash Krishnan: no disclosures

Dejana Jovanovic: Speaker fees, travel grants: Boehringer Ingelheim, Pfizer, Bayer, Sanofi Aventis. Advisory board member: Boehringer Ingelheim

Jesse Dawson: Speaker fees: Bayer, Daichi Sankyo, Pfizer, BMS, Medtronic and Boehringer Ingelheim, Astra Zeneca

Valeria Caso: Speaker fees: Boehringer Ingelheim, Pfizer/BMS, Bayer, Mindmaze, Daiichi Sankyo, Ever-NeuroPharma. Advisory board member: Boehringer Ingelheim, Pfizer/BMS, Bayer Mindmaze, Daiichi Sankyo, Ever-NeuroPharma Grants: Boehringer Ingelheim All fees of VC were paid to ARS UMBRIA.

Valeria Caso has also received funding from the European Union’s Horizon 2020 research and innovation 5.2 programme under Grant Agreement No. 754517
